# Nanostructured Gas Sensors for Medical and Health Applications: Low to High Dimensional Materials

**DOI:** 10.3390/bios9010043

**Published:** 2019-03-17

**Authors:** Noushin Nasiri, Christian Clarke

**Affiliations:** 1 School of Engineering, Faculty of Science and Engineering, Macquarie University, Sydney NSW 2109, Australia; 2 Institute for Biomedical Materials and Devices, Faculty of Science, University of Technology Sydney, Sydney NSW 2007, Australia; Christian.J.Clarke@student.uts.edu.au

**Keywords:** gas sensor, human breath, diagnosis of diseases, nano-dimensioned devices

## Abstract

Human breath has long been known as a system that can be used to diagnose diseases. With advancements in modern nanotechnology, gas sensors can now diagnose, predict, and monitor a wide range of diseases from human breath. From cancer to diabetes, the need to treat at the earliest stages of a disease to both increase patient outcomes and decrease treatment costs is vital. Therefore, it is the promising candidate of rapid and non-invasive human breath gas sensors over traditional methods that will fulfill this need. In this review, we focus on the nano-dimensional design of current state-of-the-art gas sensors, which have achieved records in selectivity, specificity, and sensitivity. We highlight the methods of fabrication for these devices and relate their nano-dimensional materials to their record performance to provide a pathway for the gas sensors that will supersede.

## 1. Introduction

Gas sensors are used in a wide variety of applications for a diverse number of industries from agriculture to environmental monitoring [1]. Among them, it is the biomedical sensor industry a market estimated to grow globally to more than 15 billion USD by 2022 that shows the most promise to take advantage of the benefits of gas sensors [2]. Compared to current standard diagnosis methods such as slow and invasive blood tests, gas sensors specifically designed for human breath analysis are an attractive alternative with real time as well as rapid and accurate diagnosis of diseases [3,4]. This ability of gas sensors to detect diseases is, in part, due to the nature of human breath, which along with containing the expected gases such as nitrogen, oxygen, water vapor, and carbon dioxide also consists of other trace species including ammonia (833 ppb), acetone (477 ppb), ethanol (112 ppb), acetaldehyde (22 ppb), and propanol (18 ppb) with some even at concentrations as low as a few parts per trillion [1,5]. When the concentrations of these trace species changes, it can be a sign of a specific disease in the case of diabetes the volatile organic compound acetone can act as a marker between healthy and diabetic patients [6,7]. Likewise, changes in the trace species of H_2_S, NH_3_, NO, and toluene can be used to diagnose halitosis, kidney malfunction, asthma, and lung cancer, respectively [8,9,10,11]. However, in order to be an effective biomedical diagnosis tool, gas sensors need to be highly sensitive to make the distinction as for example in the previous case only a <1ppm acetone concentration difference exists between the two [1,5]. However, the concentration of these biomarkers in exhaled breath may change due to a variety of parameters, such as environmental conditions or patients’ medical history [12]. In fact, the biomarkers used must be considered on a patient-to-patient basis. For instance, toluene is already found at elevated concentrations in the breath of smokers and ex-smokers [13]. However, significant differences between cancer patients, smokers, and non-smoking controls can be achieved after applying normalization [12].

**Figure 1:**
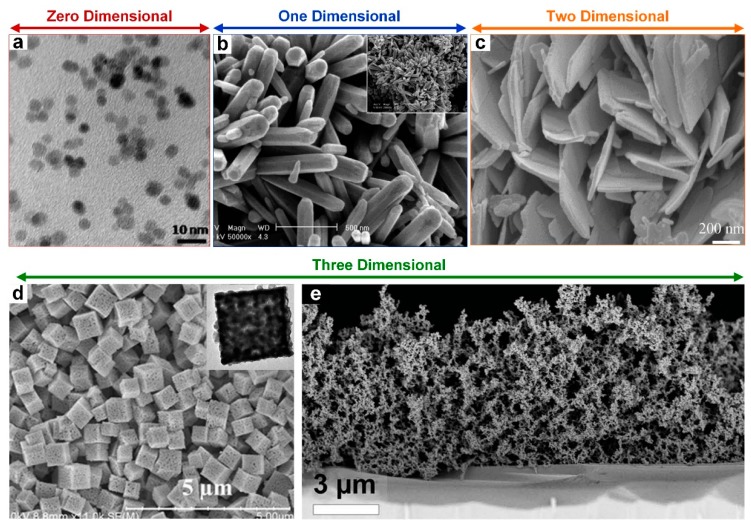
(**a**) Typical TEM image of ZnO QDs obtained through a wet synthesis method based on alkaline-activated hydrolysis and condensation of zinc acetate solutions [27]. (**b**) SEM images of ZnO nanorods bundles synthesized at 150 °C, over 4 h [28]. (**c**) SEM image of ZnO nano-sheets formed through a simple mixed hydrothermal synthesis method [29]. (**d**) SEM images of acid-washed porous SnO_2_ microcubes after calcination at 900 °C for 2 h. Inset: TEM image of the as-prepared porous SnO_2_ microcubes [30]. (**e**) ZnO ultraporous film made by flame spray pyrolysis [22].

Highly sensitive gas sensors can be produced from semiconductors such as the metal oxides ZnO [14,15], SnO_2_ [16,17], and WO_3_ [18,19], which are not only low-cost and simple to produce but also allow for a wide selection of possible nanomaterials with different nanostructured dimensions. These nanostructured dimensions highlighted in Figure 1 range from zero dimensional (0D) nanoparticles (Figure 1a), one-dimensional (1D) nanorods (Figure 1b) and nanowires (NWs), two-dimensional (2D) nanosheets (Figure 1c) and films, and three-dimensional (3D) polycrystals and ultraporous nanostructures (Figure 1d,e). These different dimensions most importantly for gas sensing affect the behavior of electrons in the nanomaterial. For instance, in 1D nanomaterials, electrons are confined in the 2D but can delocalize along the long axis whereas, for 2D nanomaterials, electrons conduct across the thickness but are delocalized in the plane of the nanomaterial [20,21]. These differences can be advantageous for the primary mechanism of a metal oxide gas sensor, which is predominantly controlled by the adsorption and desorption interaction of oxygen molecules with the target analyte [1,22,23]. Regardless of the dimension of the nanomaterial, the surface of the nanomaterial is homogenous with continuous oxygen species producing a highly electron depleted region within the nanomaterial at a distance known as the Debye length [22,24]. However, when introduced to a volatile organic compound (VOC) such as ethyl alcohol, the neutralization of those oxygen species and subsequent release of trapped electrons causes a sudden drop in resistance [25]. This mechanism is well understood and has been verified repeatedly by gas sensors produced from a range of nanomaterials with different nanostructured dimensions [8,25,26]. Each sensor upon exposure to oxygen displays a resulting acute and specific response with resistance increasing by several orders of magnitude, in the case of a ZnO film gas sensor up to 300 times [22]. The major advantage of this type of mechanism for gas sensing is its reversibility, which allows the possibility of these gas sensors to be able to regenerate for subsequent tests.

Based on this mechanism, the current state-of-the-art gas sensors for analyzing human breath have been produced by employing methods to enhance their abilities and overcome their shortcomings. These methods include introducing dopants to improve response and recovery kinetics as well as designing devices with gas sensors in arrays to allow the device greater selectivity toward target analytes [31,32,33]. Additionally, with advancements in nanofabrication, researchers have extensively combined separate nano-dimensioned materials to produce composites. The two most common composites for gas sensors are 0D/1D and 0D/2D that is the functionalizing of the surface of nanorods, nanowires, nanofilms, and nanosheets with nanoparticles [34]. These two types of composites take advantage of the large surface area of the 1D and 2D nanomaterials to produce a sensor with superior performance, which is usually related to the formation of multiple p-n heterojunctions between the two nanomaterials [15,35]. By carefully considering the band structure of each nanomaterial, a nanoscale p-n junction can be formed at the interface of the two nanomaterials. The electric field produced from these p-n junctions results in even faster response dynamics for the device since charge carriers are rapidly separated by the junctions [15,35]. These recent breath sensor development strategies have allowed sensitivity, selectivity, and response times to begin reaching the requirements for practical application and use. However, before this time, further studies will be required to overcome the final challenges posed by device integration and clinical validation [36]. 

## 2. Zero-Dimensional (0D) Gas Sensors

Zero dimensional nanomaterials including quantum dots (QDs) with the grain/particle size smaller than 10 nm have recently attracted great attention for nanoscale electronic and chemical devices due to their unique optical and electrochemical properties [37,38,39]. For example, their unsaturated bonds and high surface energies make them one of the best candidates to be used as gas sensors when low operating temperature is required [40,41]. Generally, metal oxide semiconductor films including SnO_2_, WO_3_, and ZnO can detect colorless gases such as H_2_S at significantly low concentrations [15,42,43]. However, using these metal oxide semiconductors as gas sensors requires high operating temperatures (>280 °C), which hinders their application as portable breath analysis devices [15,42,43]. In fact, most semiconductor-based gas sensors either do not operate at room temperature or demonstrate poor sensitivity to low concentrations of target analytes. Thus, it remains a major challenge to reduce the operating temperature without compromising the sensor’s performance. 

Using ultrafine nanostructures such as QDs that have a large and sensitive surface are a promising solution to decrease the operating temperature for metal oxide semiconductor-based gas sensors [38,44]. Their high surface energy allows for absorption of molecules even at room temperature. This unique property is attributed to the ultrafine size of QDs (smaller than twice Debye length), which is a key factor in the sensing performance of such devices. There are several methods for fabricating QDs such as sol-gel [45], chemical vapor deposition (CVD) [46], and self-organized growth [47]. Among them, solution processed colloidal quantum dots (CQDs) is a method that allows for fabricating and depositing solid QDs directly to substrate from the solution phase [38]. Due to its low operating temperature, this method offers substantial benefits for the fabricated QDs including a large device area, high sensitivity, ease of fabrication, and low fabrication cost [38,48]. In addition, the room-temperature fabrication of the solution processed gas sensors results in extremely small crystal size CQDs, which further enhances sensing performance [49].

**Figure 2:**
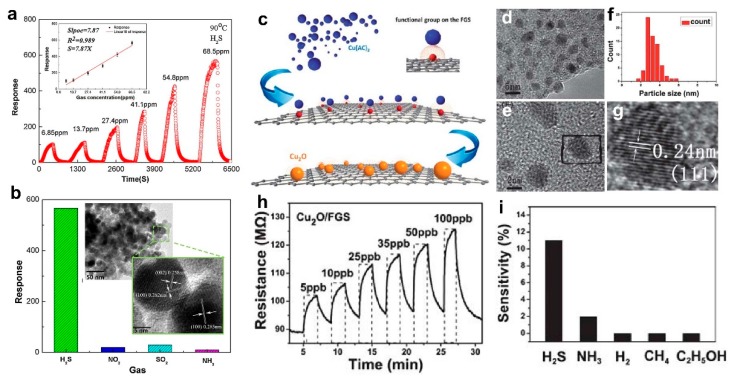
(**a**) ZnO sensing response upon exposure to H_2_S at 90 °C. Inset: the ZnO device demonstrated a linear response behavior towards H_2_S in the concentration range of 13.7 to 68.5 ppm. (**b**) Selectivity towards H_2_S with a negligible response to other gases at concentrations of 68.5 ppm. Inset: TEM images of ZnO nanoparticles with 14.6 nm diameter after annealing at 300 °C [50]. (**c**) The schematic illustration of in situ synthesis of Cu_2_O–FGS. (**d**,**e**,**g**) TEM images of Cu_2_O–FGS and (**f**) particle size distribution of Cu_2_O nanocrystals decorated on FGS. (**h**) The dynamic H_2_S sensing behavior of the Cu_2_O–FGS-based sensor device. (**i**) Cu_2_O-FGS sensitivity toward different gases: H_2_S (5 ppb), NH_3_ (25 ppm), H_2_ (25 ppm), CH_4_ (25 ppm), and C_2_H_5_OH (25 ppm) [49].

Using a colloidal procedure and spin coating method, Deng et al. [50] fabricated a super sensitive H_2_S gas sensor made of ZnO QDs of less than 4 nm in diameter. Low temperature annealing significantly enhanced the fabricated films conductivity while preserving the average grain size to below twice the Debye length. Due to this unique nanostructure morphology, the device demonstrated a high sensitivity (R_air_/R_gas_) of 75 and 567 at room temperature and low operating temperature of 90 °C, respectively, which is significantly high when compared to similar previous devices reported (Figure 2a). The fabricated ZnO QDs were deposited on an alumina substrate featuring Ag interdigitated electrodes before annealing was performed at 200 to 300 °C for 1 h. Necking between ZnO, QDs were observed after annealing at 300 °C, which leads to the QDs growth from 4 nm to 14 nm (Figure 2b, inset). Considering the room temperature Debye length of ZnO is 7.4 nm, these annealed QDs were still smaller than twice the Debye length (Figure 2b, inset). 

The device sensing performance at different concentrations of H_2_S at 90 °C is presented in Figure 2a, which demonstrates a remarkably high response of >100 to the low concentration of ca. 7 ppm [50]. The fabricated device demonstrated a great selectivity to H_2_S with negligible sensitivity to other gases such as NO_2_, SO_2_, and NH_3_ at a 68.5 ppm concentration (Figure 2b). This high selectivity is attributed to the bond energy of the H-SH bond in H_2_S (381 kJ/mol), which is less than the bond energy of other gases. When the device is exposed to the target gas, this weak bond can be easily broken during chemical adsorption, which leads to high selectivity of the device towards H_2_S gas [50]. This high selectivity combined with precise sensitivity at such low concentrations at 90 °C operating temperature make these ultrafine ZnO QDs a promising candidate for super sensitive gas sensors on wearable breath analyzers, which is a point-of-care application. However, further investigation is required to determine the accuracy and long-term stability of such devices in real world conditions [50].

Precise control over morphology and size of QDs during synthesis is a key factor in achieving high sensing performance as optical and electrical properties of QDs are drastically affected by their size. To fabricate QDs with a desired size or morphology, Zhou et al. [49] used a facile preparation method to anchor Cu_2_O QDs on functionalized graphene sheets (FGS) with precise control of the nucleation process (Figure 2c). The deposited Cu_2_O QDs were separated as single nanocrystals with no visible aggregation and were evenly distributed on the FGS surface (Figure 2d,e). The cubic QDs were about 3 nm in diameter (Figure 2f) with interplanar spacing of 0.24 nm (Figure 2g). The fabricated nanocomposite Cu_2_O/FGS gas sensor demonstrated excellent room temperature sensitivity (response of 11) towards H_2_S at the ultra-low concentration of 5 ppb, which is remarkably high when compared to the state-of-the-art devices (Figure 2h) [49]. In addition, the sensor selectivity towards H_2_S was further investigated by exposing the device to a variety of gases including NH_3_, H_2_, CH_4_, and C_2_H_5_OH (Figure 1i). Despite increasing the exposed concentration up to 25 ppm, no significant response was observed for H_2_, CH_4_, and C_2_H_5_OH when compared to the excellent response of 11 at 5 ppb for H_2_S gas (Figure 2i). In the case of NH_3_ gas, a sensing response of 2 was achieved at a concentration of 25 ppm, which is negligible compared to the device sensitivity to H_2_S (Figure 2i). This exceptional sensing performance might be attributed to the finite-size effect of the Cu_2_O QDs with their precisely-controlled crystal size of the interfacial effects between Cu_2_O and FGS that may be resulting in accelerated electronic transaction between these nanostructures [49].

**Figure 3:**
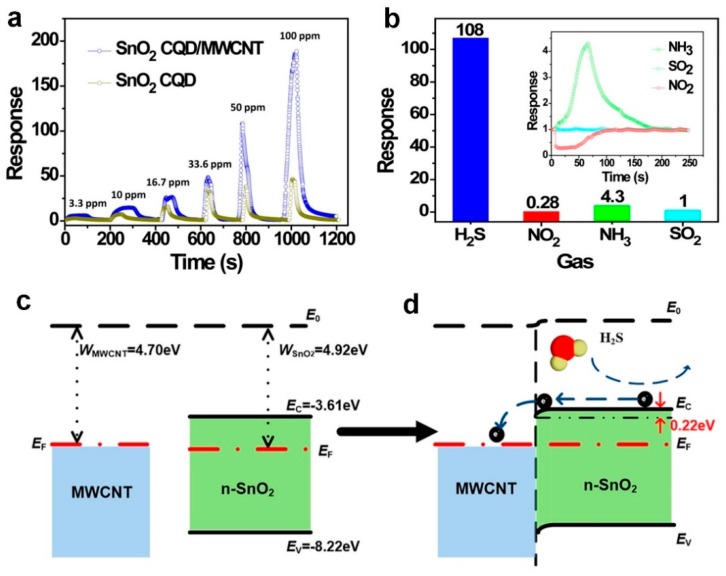
(**a**) Response of SnO_2_ CQD/MWCNT nanocomposites and pristine SnO_2_ CQDs sensors to H_2_S at 70 °C. (**b**) Selectivity of the SnO_2_ CQD/MWCNT gas sensor at 70 °C. Schematic band structure of SnO_2_ CQD/MWCNT junction: (**c**) no contact and (**d**) in contact. E_0_ denotes the vacuum level, E_F_ denotes the Fermi level, W denotes the work function, and E_C_ and E_V_ denote the conduction-band edge and valence band edge, respectively [44].

One of the major drawbacks of solution processed QDs semiconductors is their low mobility (10^−1^–10^−3^ cm^2^ V^−1^ S^−1^) [51]. Combining the excellent charge mobility of multi-walled carbon nanotubes (MWCNTs) (10^4^ cm^2^ V^−1^ S^−1^) with the outstanding molecule absorption of colloidal quantum dots (CQDs), Liu et al. [44] fabricated a SnO_2_ QD/MWCNT nanocomposite gas sensor featuring high sensitivity and selectivity towards low concentrations of H_2_S gas at the low temperature of 70 °C. Figure 3a shows a high sensor response of 5 to 3.3 ppm of H_2_S gas, which is significantly high at a low temperature of 70 °C. The theoretical limit of detection (LOD) of 43 ppb was calculated for the CQD/MWCNTs nanocomposite compared to 71 ppb for the pristine CQDs device. The sensors selectivity was investigated by comparing the sensors response to different gases including NO_2_ and NH_3_, which results in strong selectivity towards H_2_S at 70 °C (Figure 3b). This high sensitivity and selectivity could be attributed to the surface states of the fabricated QDs, electronegativity of the target gas molecules, and high mobility of the MWCNTs structure [44]. 

The band structure of the SnO_2_ CQD/MWCNT junction before and after connection is presented in Figure 3c,d. After connecting these two structures, the electrons transfer from the MWCNT to the metal oxide semiconductor QDs, which results in positive and negative charge regions at the MWCNT surface and SnO_2_ surface, respectively. When this structure is introduced to a reducing gas, such as H_2_S, the gas molecules react with the oxygen molecules adsorbed on the surface releasing oxygen trapped electrons back into the QDs conduction band [44]. This will result in an increasing number of electrons in the conduction band, and, consequently, higher conductivity of SnO_2_ QDs (Figure 3d). Benefiting from high charge mobility of the MWCNTs, the injected electrons from H_2_S to the SnO_2_ QDs could be easily transferred to the electrodes, which results in a higher sensitivity of the fabricated composite device [44].

**Figure 4:**
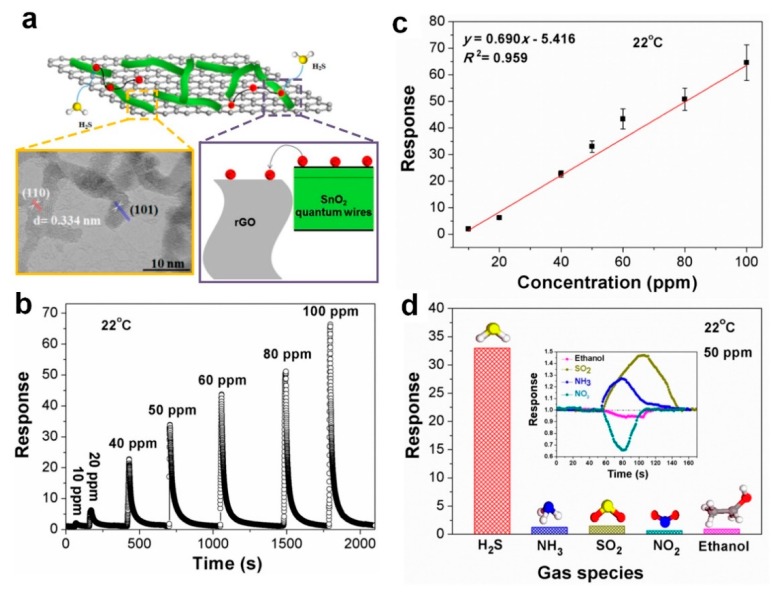
(**a**) Schematic illustration of H_2_S-sensing mechanism of gas sensors employing SnO_2_ quantum wire/rGO nanocomposites. High-resolution transmission electron microscopy (HRTEM) image of the pristine SnO_2_ quantum wires on rGO nanosheets. (**b**) Sensor response toward H_2_S gas with different concentrations from 10 to 100 ppm. (**c**) Dependence of the response upon gas concentration. (**d**) Selectivity of the optimal gas sensor employing SnO_2_/rGO nanocomposites (8 h) [52].

In another approach, Song et al. [52] fabricated a room temperature nanocomposite-based gas sensor for low concentration detection of H_2_S gas. Using a one-step spin coating method, the SnO_2_ quantum wires (QWs)/reduced graphene oxide nanocomposite (rGO) was deposited onto a ceramic substrate with QWs anchored onto the rGO nano sheets (Figure 4a). It is worth mentioning that 10 mg mL^−1^ of Cu(NO_3_)_2_ in methanol was added to the substrate (before spin coating QW/rGO solution) since Cu can act as a catalyst for SnO_2_-based sensors. Combining the fast electron transport kinetics of the rGO 2D structure with the high specific surface area of QWs, the fabricated nanocomposite sensor features a high response of 33 towards a 50 ppm concentration of H_2_S at 22 °C (Figure 4b). A 43-ppb limit of detection was calculated at 22 °C with the sensor response of 1.02, which is still high for such a low operating temperature (Figure 4c). The device selectivity was further evaluated, which results in excellent H_2_S selectivity against other gases including NH_3_, SO_2_, and NO_2_ (Figure 4d). This high selectivity might be attributed to the presence of copper, which is known to enhance sensor device performance by acting as a catalyst or forming hetero-junctions [52]. 

## 3. One Dimensional (1D) Gas Sensors

One dimensional (1D) nanomaterials including nanowires, nanofibers, nanorods, and nanotubes have attracted great attention for sensing applications due to their unique morphology and high surface-to-volume ratio [20,21]. Among different 1D nanostructures, nanotubes have attracted great attention as promising nanostructures for fabricating highly sensitive and selective gas sensors due to their vast surface area and open porous structure [53]. In fact, the meso and nanosized pores formed on various nanotubes surfaces during synthesis can significantly enhance the gas sensing performance by facilitating the penetration of targeting gas into the deepest parts of the sensing device.

**Figure 5:**
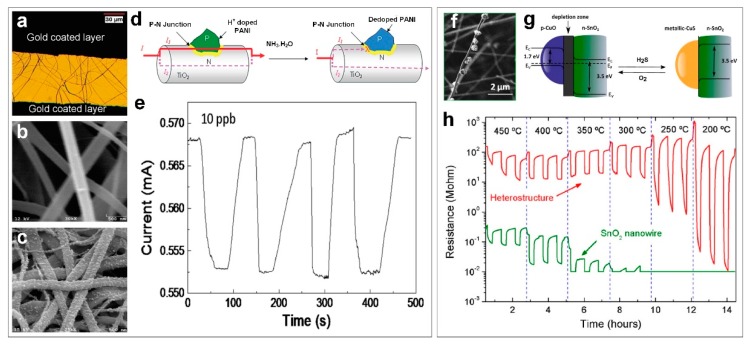
(**a**) An optical microscope image of the fabricated CuO/SnO_2_ sensor and (**b**) high-magnification scanning electron microscopy images of TiO_2_ microfibers and (**c**) TiO_2_ microfibers enchased with PANI nanograins. (**d**) Schematic of nanosized p-n heterojunction as a switch to control the electric current flow in TiO_2_ microfibers. (**e**) Sensor’s reproducibility upon exposure to 10 ppb NH_3_ gas [54]. (**f**) SEM image of p-CuO particles onto a single SnO_2_ nanowire. (**g**) General sketch of the heterostructure: p-CuO particles on the n-SnO_2_ surface create a p-n junction. The depleted region reduces the effective conduction channel in the nanowire, which leads to a higher resistance. After H_2_S sensing, p-CuO particles change to metallic CuS, which result in a breakup of the p-n junction, a wider conduction channel in the nanowire, and, as a consequence, a lower resistance value. (**h**) Response of p-CuO/n-SnO_2_ heterostructures and bare SnO_2_ nanowires to 2 ppm H_2_S at different temperatures [42].

Using a combination of conducting polymer (polyaniline) with inorganic nanostructured material (TiO_2_ fibers), Gong et al. [54] fabricated a p-n junction-based gas sensor (Figure 5a) capable of detecting ultra-low concentrations of NH_3_ (10 ppb) at room temperature (Figure 5e), which is 1000-times more sensitive when compared to the state-of-the-art pure polyaniline sensors. This significant enhancement could be attributed to the unique conductivity of the polyaniline polymer in this sensor as a function of H^+^ doping degree in addition to the excellent selectivity of the TiO_2_ fibers [54]. Figure 5 shows the scanning electron microscopy (SEM) images of the TiO_2_ fibers before (Figure 5b) and after (Figure 5c) coating with polyaniline nanograins. The device sensing mechanism is illustrated in Figure 5d. A p-n junction is built at the interface of the p-type polymer and the n-type metal oxide semiconductor (Figure 5d). As H^+^ doped polyaniline is conductive, the polymer coated TiO_2_ fibers demonstrated a higher conductivity compared to pure TiO_2_ fibers when the applied voltage is merely above the breakdown voltage [54]. Then, in the case of NH_3_ exposure, the polymer particles on the TiO_2_ surface will become de-doped, which results in a rapid decrease in device conductivity (Figure 5d).

In a similar approach [42], a p-n heterostructure sensor for selective detection of H_2_S gas was fabricated using p-type CuO nanoparticles and n-type SnO_2_ nanowires (Figure 5f). Using a two-step chemical vapor deposition (CVD), SnO_2_ nanowires were initially deposited on Al_2_O_3_ substrates featuring pre-patterned Pt electrodes. CuO nanoparticles were then deposited on pre-grown SnO_2_ nanowires (Figure 5f), which formed a p-n hetero-structure device after air annealing at 300 °C for 24 h [42]. The fabricated device demonstrated an increase in sensitivity toward H_2_S gas, from 9 of the pure SnO_2_ to 3261 for the p-n heterostructure device (Figure 5h) while negligible change in sensing performance was observed between the pure SnO_2_ and the heterostructure sensor, when exposed to CO and NH_3_ gases [42]. This enhanced sensing performance is attributed to the unique configuration of the device. In fact, due to the respective Fermi level positions of CuO and SnO_2_, the CuO/SnO_2_ configuration amplified the device selectivity when exposed to H_2_S gas (Figure 5g, left). To compliment this, CuO is converted to CuS (with metallic characteristics) when it is exposed to H_2_S gas, which leads to a significant change in the device structure, from p-n heterostructure to metal-semiconductor configuration (Figure 5g, right). The Fermi level positions in the device configuration also play a significant role in the electrical properties (Figure 5g), which results in remarkably higher sensing performance (both sensitivity and selectivity) when compared to pure metal oxide semiconductors (Figure 5h).

**Figure 6:**
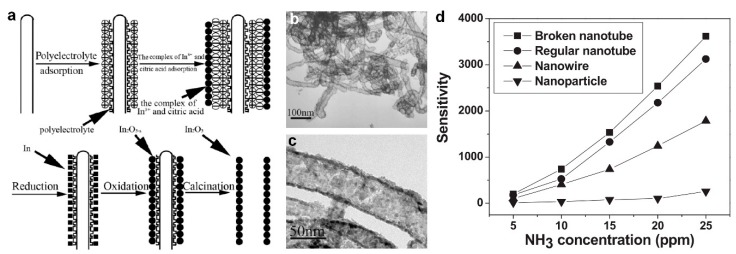
(**a**) Schematic diagram for the growth process of In_2_O_3_ nanotubes. (**b**,**c**) TEM images of regular In_2_O_3_ nanotubes prepared by the calcination of In_2_O_3_/polyelectrolyte/CNT nanocomposites at 550 °C in O_2_ for 3 h. (**d**) Sensitivity response versus ammonia concentration (5–25 ppm) at room temperature for four types of gas sensors based on In_2_O_3_ nanostructures including broken In_2_O_3_ nanotubes, regular In_2_O_3_ nanotubes, In_2_O_3_ nanowires, and In_2_O_3_ nanoparticles [55].

In addition to commonly used semiconductors such as ZnO, SnO_2_, and TiO_2_, In_2_O_3_ nanotubes have attracted great attention for a gas sensing application because of their impressive electrical properties as well as ultra-high surface-to-volume ratio. Using carbon nanotubes (CNTs) as an ideal template, Du et al. [55] fabricated porous In_2_O_3_ nanotubes as superior gas sensing candidates for detecting NH_3_ at room temperature. Figure 6a illustrates the schematic for the fabrication process, using a layer-by-layer assembly technique to form a polyelectrolyte on the surface of the CNTs. Following reduction and oxidation reactions on the surface of the CNTs, porous In_2_O_3_ nanotubes were obtained by calcination and removal of the CNT template in O_2_ at 550 °C for 3 h [55]. Transmission electron microscopy (TEM) images of the fabricated porous with a 30 to 60 nm diameter In_2_O_3_ nanotubes are presented in Figure 6b,c. Higher magnification TEM investigation (Figure 6c) revealed the presence of nanopores on the nanotube’s wall, which formed during the removal of the CNTs by the calcination process. These nanometer-sized pores significantly increased the surface-to-volume ratio of the fabricated In_2_O_3_ nanotubes, which results in higher gas sensing performance toward NH_3_ gas [55].

In order to investigate the device sensitivity toward NH_3_ gas and indicating the optimal nanostructure, four different types of In_2_O_3_ gas sensors were fabricated and tested between NH_3_ gas concentrations of 5 to 25 ppm. The four types included broken nanotubes, which were the result of an ultra-thin primary coating layer of amorphous In_2_O_3_, regular nanotubes presented in Figure 6b,c, nanowires fabricated by a thermal evaporation method, and nanoparticles fabricated via layer-by-layer assembly without the CNT template [55]. As shown in Figure 6d, the broken nanotubes demonstrated the highest sensitivity of 3800 at room temperature for 25 ppm NH_3_ gas, which was about 20 times higher than In_2_O_3_ nanoparticles sensitivity. This significantly higher sensing performance could be attributed to the higher surface-to-the-volume ratio of the broken nanotubes compared to that of In_2_O_3_ nanoparticles [55]. 

**Figure 7:**
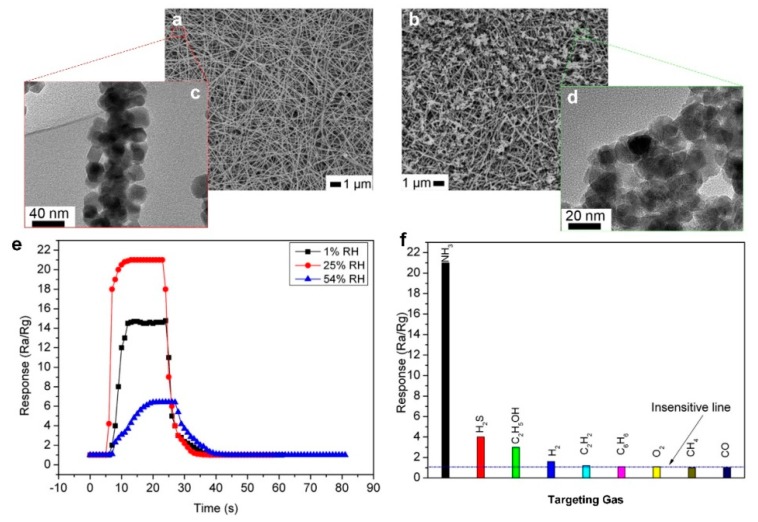
SEM images of (**a**) pure In_2_O_3_ nanofibers and (**b**) 16at% SnO_2_ nanoparticles/In_2_O_3_ nanofibers. The TEM images of (**c**) SnO_2_ nanoparticles and (**d**) In_2_O_3_ nanofibers. (**e**) Response-time curves of 16at% SnO_2_/In_2_O_3_ nanofiber sensors to (**e**) 1 ppm NH_3_ at 1%, 25%, and 54% RH and (**f**) 1 ppm of different gases at room temperature [56].

In a similar approach, Qi et al. [56] coated electrospun In_2_O_3_ nanofibers (Figure 7a,c) by SnO_2_ nanoparticles (Figure 7b,d), which demonstrated an excellent sensing response of 21 for NH_3_ detection at room temperature. The sensor showed a high sensing response of 2 at low gas concentrations of 100 ppb, which is significantly higher than the sensitivity required for detecting kidney disease by analyzing human breath [57,58]. The fabricated device demonstrated specific selectivity toward NH_3_ gas, with 7-times and 5-times lower sensitivity to acetone (C_2_H_5_OH) and H_2_S, respectively, and no sensitivity to H_2_, C_2_H_2_, C_6_H_6_, O_4_, CH_4_, and CO (Figure 7f). This performance could be attributed to the extra absorbing sites provided by SnO_2_ nanoparticles on the In_2_O_3_ nanofiber surface, which results in a high sensing response (R = 22) and a fast response dynamic (about 10 s) for detecting 1 ppm of NH_3_ at relative humidity of 25% (Figure 7e). However, a high sensitivity for the relative humidity of human breath (~85%) is required for point-of-care application.

**Figure 8:**
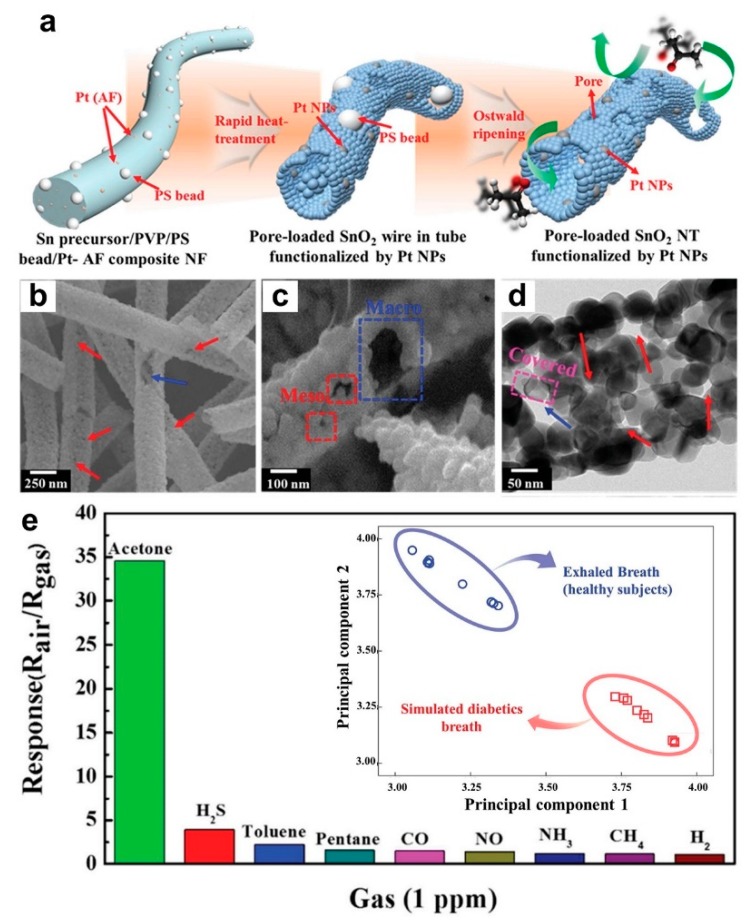
(**a**) Schematic illustration of the Sn precursor/PVP/PS bead/Pt@AF composite NF (left), pore loaded SnO_2_ wire in tube functionalized by Pt NPs (middle), and pore loaded SnO_2_ NTs functionalized by Pt NPs (right). (**b**,**c**) Porous SnO_2_ NTs (PS_SnO_2_ NTs) and (**d**) TEM image of PS_SnO_2_ NTs. (**e**) Selective characteristics of Pt-PS_SnO_2_ NTs at 350 °C. Inset: Pattern recognition based on PCA using sensor arrays (PS_SnO_2_ NTs, Pt-PS_SnO_2_ NTs, and Pt-SnO_2_ NTs) [6].

In another approach, a single nozzle electrospinning method was used to synthesize Sn precursor/polyvinylpyrrolidone (PVP) composite nanofibers with uniformly distributed polystyrene (PS) and Pt@AF (Pt NPs encapsulated in apoferritin) colloidal beads (Figure 8a, left) [6]. The PVS matrix (and PS beads), which was used as the template for the nanofibers, were then removed through a calcination step at 600 °C for 1 h, which leads to the formation of Pt decorated SnO_2_ nanofibers with macro pores (Figure 8a, middle) and meso pores (Figure 8a, right) on the surface [6]. Figure 8b–d presents the microscopic images of fabricated Sn precursor/PVP composite nanofibers (Figure 8b) and porous SnO_2_ nanotubes (after calcination) (Figure 8c,d) with macro-pores and meso-pores formed on their walls. A 0.08 wt.% of Pt was found to be the optimal Pt catalyst concentration for these porous SnO_2_ nanotubes to achieve the highest sensor response to acetone (with a concentration of 100 ppb to 5 ppm) at the operating temperature of 350 °C and relative humidity of 90% [6]. In fact, the fabricated sensor demonstrated a high sensitivity of 4.3 to a low acetone concentration of 100 ppb, which indicates an excellent LOD of 10 ppb at such a high relative humidity [6].

In addition, the fabricated device displayed outstanding selectivity toward acetone (34.8 at 1 ppm) with negligible response to other gases including H_2_S, C_7_H_8_, C_5_H_12_, CO, NO, NH_3_, CH_4_, and H_2_ (Figure 8e) [6]. This high sensing performance is mostly attributed to the optimal wt.% of the Pt catalyst, which works as a chemical sensitizer, separating C-H, O-H, and O_2_ bonds, increasing the device selectivity towards acetone. The sensing mechanism could be ascribed to both the highly porous nanostructure as well as the sensitization effect of uniformly dispersed Pt NPs on the SnO_2_ nanotube surface [6]. Furthermore, a nanoscale p-n junction was formed on the wall of the n-type SnO_2_ nanotubes after depositing p-type Pt nanoparticles, which resulted in a significantly larger depleted region compared to the pure SnO_2_ nanotubes. This leads to a higher baseline resistance for the former nanostructured device. In the case of acetone exposure, the PtO nanoparticles on the SnO_2_ nanotube surface donate electrons to SnO_2_ through a reduction reaction (PtO reduces to Pt), which results in a smaller depleted region and, consequently, a higher resistance change in the fabricated device [6].

The sensing properties of the Pt NPs/SnO_2_ nanotubes were further investigated by exposing the device to simulated diabetic breath as well as exhaled breath of healthy subjects (10 breath samples for each group). The collected data was then analyzed using a principal component analysis (PCA) to distinguish the diabetics breath from the healthy subjects (Figure 8e, inset). With no overlaps in the data, it demonstrates that these fabricated nanostructures show great promise to be utilized as portable breath sensors for disease diagnosis [6].

In a similar work, Kim et al. [59] reported fabricating electrospun mesoporous WO_3_ nanofibers (with diameter of about 445 nm) functionalized with PtM nanoparticles (where M could be either Pd, Rh, or Ni), which demonstrates excellent performance in detecting acetone and H_2_S at ppb concentration levels and high relative humidity of 90%. This is similar to human exhaled breath. The PtM nanoparticles were synthesized by mixing Pt^+^ and other metal ions (Pd^2+^, Rh^2+^, and Ni^2+^) in a single apoferritin shell, which results in the formation of spherical PtPd, PtRh, and PtNi nanoparticles with an average particle size of 2 to 3 nm [59]. 

**Figure 9:**
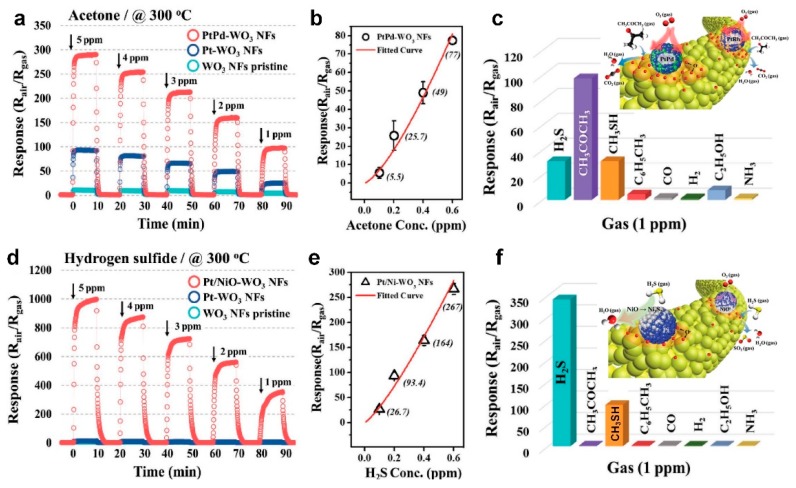
Dynamic gas-sensing properties of pristine WO_3_ NFs, Pt-, PtPd-, PtRh-, and Pt/NiO-WO_3_ NFs. Acetone sensing property of the PtPd-WO_3_ NFs at 300 °C: (**a**) response property in concentration ranges of 1 to 5 ppm, (**b**) limit of detection, (**c**) selective property toward 1 ppm as well as the response towards seven interfering gases: hydrogen sulfide (H_2_S), methyl mercaptan (CH_3_SH), toluene (C_6_H_5_CH_3_), carbon monoxide (CO), hydrogen (H_2_), ethanol (C_2_H_5_OH), and ammonia (NH_3_). Hydrogen sulfide sensing property of the Pt/NiO-WO_3_ NFs at 300 °C: (**d**) response property in concentration ranges of 1 to 5 ppm, (**e**) limit of detection, and (**f**) selective property toward 1 ppm as well as the response toward seven interfering gases [59].

Figure 9a,d present the dynamic sensing response of fabricated WO_3_ nanofibers upon exposure to acetone and H_2_S, respectively, with a concentration range of 1 to 5 ppm [59]. In the case of acetone detection, the PtPd-WO_3_ nanofibers demonstrated the highest sensing performance with the sensor response of 97.5 at 1 ppm and 300 °C (Figure 9a), which was more than 20 times higher than pure WO_3_ nanofibers (4.3 at 1 ppm) and significantly higher than the state-of-the-art. The fabricated device featured an exceptional LOD of 1.07 ppb (Figure 9b), which is the lowest LOD reported in literature for such semiconductor-based sensors [59]. It is worth mentioning that the PtPd loading amount was only 0.0084 wt.%, but greatly enhanced the sensing performance of the fabricated fibers. The device selectivity was further investigated upon exposure to 1 ppm of different gases including CH_3_COCH_3_, H_2_S, CH_3_SH, C_6_H_5_CH_3_, CO, H_2_, C_2_H_5_OH, and NH_3_ (Figure 9c). The PtPd-WO_3_ nanofibers demonstrated an outstanding selectivity toward acetone (response of 97.5 at 1 ppm) with minimal response upon exposure to H_2_S and CH_3_SH (response of ca. 30 at 1 ppm) and negligible response to other gases (Figure 9c) [59]. 

In the case of H_2_S detection, Pt/NiO-WO_3_ nanofibers demonstrated a significant enhancement in sensing performance with exceptional sensor response of 340 to 1 ppm of H_2_S gas, compared to the response of 3.77 for pure WO_3_ nanofibers (Figure 9d). The Pt/NiO-WO_3_ nanofibers response rose up to 1000 for higher H_2_S concentrations (5 ppm) with excellent LOD of 54 ppt for H_2_S detection (Figure 9e). The fabricated Pt/NiO-WO_3_ nanofiber device featured a great selectivity toward H_2_S (response of 340 at 1ppm) with a low response to CH_3_SH (response of 75 at 1ppm) and only minimal response upon exposure to other gases (Figure 9f) [59].

The higher sensing performance of these fabricated nanofiber devices is mostly attributed to the mesoporous structure of the semiconductor metal oxide nanofibers facilitating the penetration of target gases into the deepest layers of the sensor, as well as the catalytic effect of functionalized PtM nanoparticles deposited on the semiconductor metal oxide nanofiber surface [59]. The decorated catalytic nanoparticles accelerate the oxygen molecules dissociation on the nanofiber surface through a spill-over effect, which results in an enhanced number of oxygen molecules chemisorbed on semiconductor metal oxide surface (Figure 9c,f, insets). Upon exposure to the targeting gas, the catalytic nanoparticles ensure an enhanced rate of reaction between chemisorbed oxygen and the targeting gas molecules, which leads to a greater resistance change in the fabricated sensing device (Figure 9c,f, insets) [59]. 

## 4. Two-Dimensional (2D) Gas Sensors

**Figure 10:**
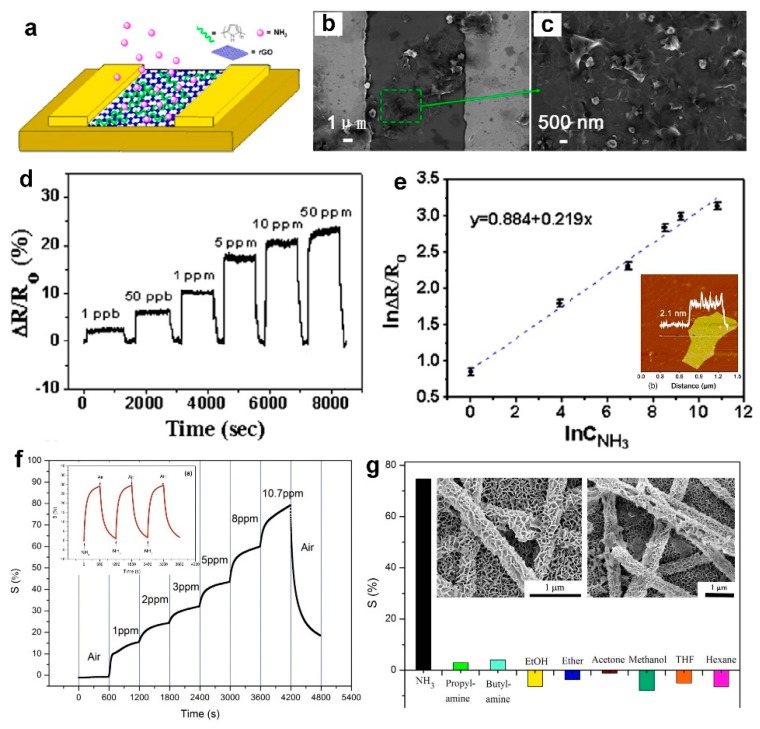
(**a**) Schematic diagram of the gas sensors based on rGO. (**b**) SEM image of a sensing device composed of rGO platelets that bridge neighboring Au fingers. (**c**) An enlarged image of rGO membrane formed between electrodes. (**d**) Plot of normalized resistance change versus time for the sensing device based on rGO upon exposure to NH_3_ gas with concentrations ranging from 1 ppb to 50 ppm. (**e**) Log–log plot of response variation of the rGO sensor as a function of NH_3_ concentration [62]. (**f**) Dynamic responses of SnO_2_/PPy nanocomposite to different NH_3_ concentrations at room temperature. Inset: Sensor’s response to 5 ppm of NH_3_ at room temperature. (**g**) Response magnitude of SnO_2_/PPy nanocomposite to different vapors at room temperature. Concentration of the vapors: [NH_3_] = 10.7 ppm. [Propyl amine] = [butyl amine] = 10 ppm. 5000 ppm for other organic solvents. Inset: SEM images of electrospun nanofibers before (left) and after hydrothermal treatment at 135 °C for 8 h [65].

Two-dimensional materials have received attention in recent years for gas sensing applications due to their unique chemical and physical properties such as high electron conductivity and excellent mechanical strength caused by their quantum and surface effects [21,60]. Many 2D materials such as graphene, graphene oxide, MoS_2_, and WS_2_ have been widely used for trace detection of analytes and exhibited great potential for gas sensing applications due to their high surface to volume ratio [61,62,63,64]. Among them, reduced graphene oxide (rGO) is a promising candidate for gas sensing devices due to its outstanding electrical and thermal properties with a high solution processability compared to graphene [52,62]. Hu et al. [62] used a simple extraction and micro-syringe deposition method (drop-drying) to fabricate rGO based sensors (Figure 10a) consisting of networks on rGO platelets (with average thickness of 2.1 nm) (Figure 10e, inset) covering Cr and Au electrodes (Figure 10a–c). The fabricated device demonstrated ultra-sensitive and ultra-fast sensing response upon exposure to NH_3_ gas with different concentrations from 50 ppm down to 1 ppb (Figure 10d). As presented in Figure 10d, a considerably high response of 2.4% was achieved even at a very low NH_3_ concentration of 1 ppb, which was the highest response reported for such an ultralow concentration [62]. The device repeatability was further investigated by exposure to four cycles of NH_3_ gas at a concentration of 5 ppm, which results in a constant response of 17% with fast response dynamics. This demonstrates a great stability and repeatability of the fabricated device toward NH_3_ [62].

In another study, Li et al. [65] proposed a novel method for the in-situ growth of SnO_2_ nanosheets (Figure 10g, inset) through hydrothermal treatment of electrospun SnO_2_ nanofibers on ceramic substrates featuring interdigitated Au electrodes. The nanocomposite SnO_2_/polypyrrole (PPy) nano-sheets were then fabricated by adding a PPy layer on the nanosheets using a vapor phase polymerization technique. The sensing response of the fabricated SnO_2_/PPy nano-sheet composite sensors upon exposure to NH_3_ is presented in Figure 10f. The sensing performance of the fabricated device increased from 15% for 1 ppm to 80% for 10.7 ppm of NH_3_ gas at room temperature, with no saturation behavior at higher concentrations of the targeting gas, which proposes these 2D nano-sheet composites as a promising nanostructure for NH_3_ detection over a wide range of concentrations (Figure 10f) [65]. This high sensitivity could be attributed to the formed p-n junction between p-type PPy and n-type SnO_2_. In addition, the device demonstrated a great repeatability toward NH_3_, with stable but slow dynamic response over air/NH_3_ test cycles with response and recovery times of 259 and 468, respectively. Furthermore, the device exhibited an excellent selectivity toward NH_3_ (with response of ca. 75% to 10.7 ppm NH_3_) than other gases including ethanol, acetone, and hexane. The concentration of organic gases including ethanol and acetone was about 500 times higher than NH_3_ concentration (5000 ppm vs 10.7 ppm) [65].

**Figure 11:**
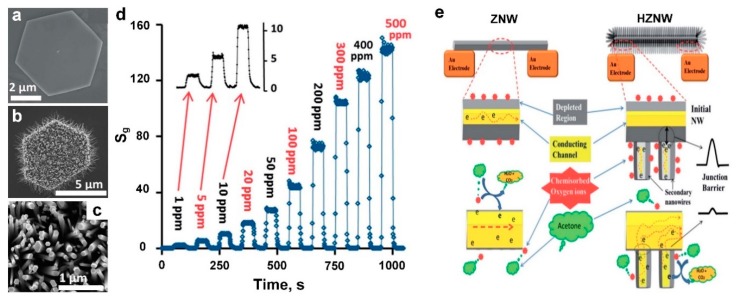
SEM image of single (**a**) ZND, (**b**) HZND, and (**c**) the secondary NWs. (**d**) Response vs. time curve of the HZND sensor in the range of 1 to 500 ppm under the optimum conditions of 425 °C. (**e**) A schematic diagram of the sensing mechanism of ZnO NW vs. hierarchical nanostructures: charge transport in single NW (left) and the effect of the secondary NW–initial nanostructure junctions (right) on the charge transport in the hierarchical nanostructures [21].

Aiming for an optimal nanostructure with high surface-to-volume ratio, Alenezi et al. [21] combined 1D ZnO nanowires with 2D ZnO nano-discs to develop a hybrid hierarchical architecture with a large surface-to-volume ratio, which featured a targeted sensing performance toward acetone gas, which is a well-known biomarker for diabetes. Figure 11a–c show the SEM images of a 2D ZnO nano-disc (ZND) (Figure 11a), hierarchical ZnO nanowires (HZNWs) (Figure 11b), and hierarchical ZnO nano-disks (HZNDs) (Figure 11c) synthesized through a two-step seeded growth approach. Using ZnO nano-disks (ZNDs) as seeds, the ZnO nanowires (ZNWs) grow along [1] covering the entire 2D surface, which results in the synthesis of 3D HZNDs, with high density and uniformity of the secondary NWs (Figure 11b,c) [21].

The device sensing performance upon exposure to acetone was investigated at different operating temperatures with an optimal temperature found to be 425 °C for a sensing response of 42 to 100 ppm (Figure 11d). The fabricated HZNDs demonstrated a higher sensing response to the targeting gas when compared to the initial 2D ZNDs and ZNWs [21]. In addition, the sensing dynamic was improved for HZNDs with response and recovery time of 2 seconds and 4 seconds upon exposure to 100 ppm acetone, which was ca. 4–5 times faster compared to the pure ZNWs structure (10 and 15, respectively) [21]. However, decreasing the acetone concentration down to 20 ppm led to a significant increase in the response time of the fabricated device (5 s), which could be related to the mean residence period of the acetone molecules on the HZNDs surface. A high response of 3 was achieved for a concentration of 1 ppm (Figure 11d, inset), which is useful for medical application and diagnosing diabetes [21].

The sensing principle of single ZNW as well as HZNWs is presented in Figure 11e. As mentioned before, oxygen plays a significant role in metal oxide semiconductor-based devices where the detection mechanism is mainly based on oxygen adsorption on the surface capturing free electrons from the conduction band and forming electron depleted regions [21]. Upon exposure to a reducing gas, such as acetone, the acetone molecules are oxidized on the surface by the adsorbed oxygen, releasing electrons to the conduction band and decreasing the device resistance (Figure 11e, left). In the case of the HZNW/HZNDs nanostructure, secondary nanowires act as junction barriers, hindering the electron transfer from one ZNW to another and improving the sensing performance of the fabricated hierarchical device (Figure 11e, right) [21]. In addition, the number of surface defects in the hierarchical nanostructures is significantly higher when compared to the initial ZNWs, which can be considered as extra junction barriers that result in a higher sensing response in the HZNDs nanostructure [21].

## 5. Three-Dimensional (3D) Gas Sensors

Porous nanostructured films are the most common morphologies for nanoscale gas sensors due to their high specific surface area and low power consumption [15,18,66]. In addition, the ability for 3D nanostructured devices to be made into layers of porous thin films comprised from differently selected materials can potentially be exploited to introduce a single sensor to detect a wide spectrum of sensing targets [67].

**Figure 12:**
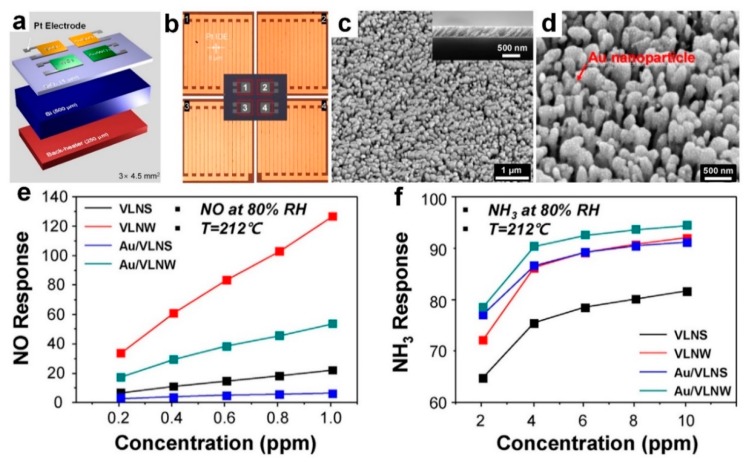
(**a**) Schematic diagrams of chemi-resistive electronic nose (CEN) with micro-heater on the back-side). (**b**) Optical images of four Pt interdigitated electrodes (IDEs) with spacing and width of 5 and 40 m, respectively. (**c**,**d**) Top-view and cross-sectional FE-SEM images (inset) of VLNs of WO_3_ (VLNW) and Au-functionalized VLNW (Au/VLNW). Sensing response of each channel in the CEN as a function of (**e**) NO and (**f**) NH_3_ concentration at 212 °C in 80% RH condition [68].

Using an electron beam evaporation in a glancing angle deposition, Moon et al. [68] fabricated an array of villi-like nanostructured (VLN) chemi-resitive sensors made of metal oxide semiconductors including villi-like nanostructured SnO_2_ (VLNS) and villi-like nanostructured WO_3_ (VLNW) (Figure 12a,b). The top-view and cross-sectional FE-SEM images of the fabricated villi-like WO_3_ after Au functionalization (with Au nanoparticles of 50 nm in diameter) are presented in Figure 12c,d, respectively. The fabricated device demonstrated a closely packed nanostructure with high film porosity resulting in a high response of 133 and 20 towards NO and NH_3_ gases, respectively, at the operating temperature of 212 °C and 80% RH (Figure 12e,f). This high sensing performance is mainly attributed to a large surface-to-volume ratio of these porous nanostructures (Figure 12c,d), which leads to an affective gas diffusion throughout the film as well as spillover effect from the Au nanoparticles [68].

In fact, functionalizing these metal oxide semiconductors with Au nanoparticles hindered the adsorption of water molecules on the surface due to an increase in the number of O- ions. The device demonstrated a LOD of 899 ppt and 312 ppb towards NO and NH_3_ gases, respectively, which are significantly lower than the detection limit required for disease detection including asthma (50 ppb NO) and kidney failure (830 ppm NH_3_) [10,68,69]. In addition to this excellent sensitivity, the fabricated VLNW demonstrated a specific selectivity towards NO with negligible sensing response to the other gases (Figure 12g), which could be attributed to their high stability and insufficient thermal energy to react with other molecules [68]. For example, the covalent bond between carbons in C_7_H_8_ and C_6_H_6_ benzene rings hinders their reaction with other molecules due to their high chemical stability [68].

**Figure 13:**
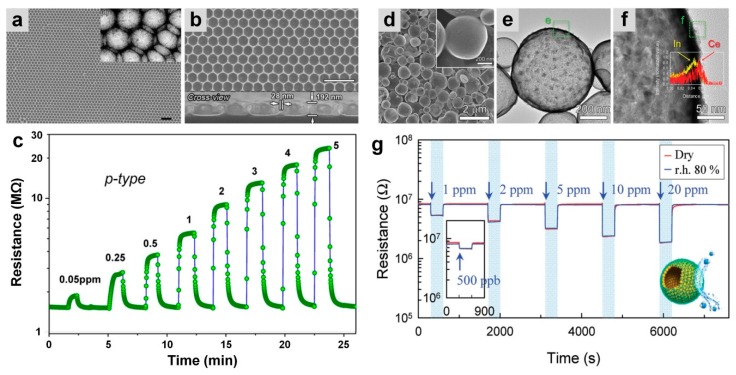
(**a**) Typical SEM image of the LaFeO_3_ ordered porous film and the corresponding TEM image (inset). (**b**) High-magnification image of a, and the corresponding cross view (inset). (**c**) Dynamic sensing response of the fabricated device upon exposure to 0.05 to 5 ppm of ethanol [70]. (**d**) SEM and (**e**) TEM images of 11.7 Ce-In_2_O_3_ hollow spheres with (**f**) elemental mapping. (**g**) Dynamic sensing transients of 11.7 Ce-In_2_O_3_ hollow spheres exposed to 0.5 to 20 ppm of acetone at 450 °C in dry (red) and r.h. 80% (blue) [74].

In another approach, Dai et al. [70] fabricated an LaFeO_3_ perovskite porous thin film using a facile template-transferring and solution-dipping strategy, which results in a monolayer honeycomb-like porous nanostructure (Figure 13a,b) with a single phase of perovskite. The high-quality colloidal sphere monolayer with good homogeneity combined with high porosity of this nanostructure resulted in an enhanced sensing performance for the LaFeO_3_ porous film in comparison to the dense bulk film [70]. Figure 13a,b show the structural properties of the fabricated porous films, which demonstrate crack-free and homogenous honeycomb-based arrays (Figure 13a, inset), with 28 nm thick and 192 nm high pore walls (Figure 13b). 

The fabricated LaFeO_3_ film revealed a typical p-type behavior upon exposure to reducing gases with superior selectivity toward ethanol (response of 15 to 5 ppm) than other gases including acetone (response of 4.5) and CO (response of ca. 2.5) at a temperature of 450 °C. A 50 ppb LOD was achieved for this porous thin film with the sensor response of 1.22 toward ethanol in the range of a few seconds (response and recovery time of 6 and 5 s, respectively) [70]. 

The greatest drawback for such oxygen dependent sensors is the reaction between water vapor molecules with oxygen ions on the semiconductor metal oxide surface, which results in the formation of less reactive hydroxyl groups and, consequently, less sensing performance [16,71]. Several solutions have been suggested to overcome this obstacle including increasing the operating temperature and doping with NiO and CuO that have high affinity to water molecules [71,72,73]. Recently, Yoon et al. [74] reported a new method for fabricating a humidity-independent In_2_O_3_ gas sensor, made of hollow spheres of In_2_O_3_ (by ultrasonic spray pyrolysis) (Figure 13d) coated by different concentrations of CeO_2_ nanoclusters (Figure 13d–f) using a layer-by-layer (LBL) deposition technique. Figure 13e,f present the 5 nm in diameter CeO_2_ nanoclusters deposited in the surface of the hollow In_2_O_3_ spheres [74]. In the case of pure In_2_O_3_, the device demonstrated a typical characteristic n-type semiconductor response to humidity with the sensing response of 22.2 and 4.76 in a dry and 80% RH atmosphere, upon exposure to acetone [74]. While the In_2_O_3_ sensor coated by CeO_2_ nanoclusters demonstrated the same sensing responses in both dry and 80% RH atmospheres, an LOD of 500 ppb resulted due to an acetone interaction (Figure 13g). 

**Figure 14:**
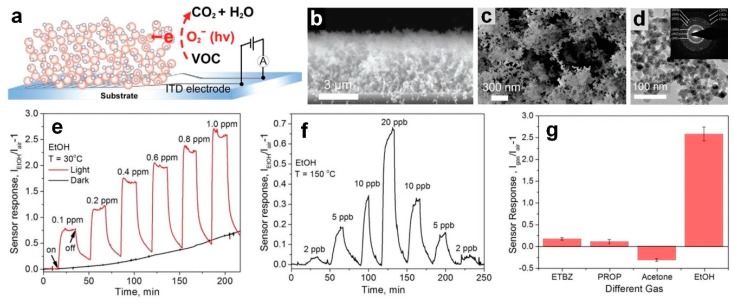
(**a**) Schematic representation and (**b**,**c**) SEM images of the p–n NiO–ZnO nanoheterojunctions devices for chemoresistive sensing of VOC. (**d**) TEM image of the fabricated NiO-ZnO nanoparticles with inset SAED pattern. (**e**) Dynamic responses of 1 nm NiO–6 µm ZnO nanoheterojunction film to ethanol molecules as a function of its concentration from 0.1 to 1 ppm at room temperature in dark or under solar irradiation. (**f**) Dynamic responses of a 1 nm NiO–ZnO film to ethanol with concentrations from 2 to 20 ppb at 150 °C under solar irradiation. (**g**) Sensor response of a 1 nm NiO–ZnO film to 1 ppm of ethylbenzene, propane, acetone, and ethanol at 30 °C under solar irradiation [15].

Very recently, Chen et al. [15] fabricated a nano-heterojunction layout consisting of a 3D ultra-porous network of NiO (p-type)/ZnO(n-type) semiconductors (Figure 14a) for the rapid room temperature sensing of ethanol. The ultra-porous ZnO nanostructured film was deposited on a glass substrate featuring interdigitated Pt electrodes, using a flame spray pyrolysis system and resulting in a 6 m thick ZnO film with 98% porosity (ZnO mass thickness of ≈120 nm). Due to the high porosity of this ZnO film, the sputtered NiO nanoclusters could penetrate to the deepest layer of the ZnO and form a homogeneous p-n heterojunction nanostructured device (Figure 14b,c). A TEM image of spherical ZnO nanoparticles (20 nm in diameter) coated with NiO clusters collected from the glass substrate is presented in Figure 14d. The dynamic response of the fabricated p-n heterojunction device (with 1 nm NiO/6 μm ZnO) toward ethanol is presented in Figure 14e, in dark and under light illumination (AM1.5 solar simulator, 67 mW cm^−2^). At room temperature and in the dark, no response was observed from pure ZnO upon exposure to ethanol. The fabricated sensor response significantly increased under solar irradiation [15]. In fact, the formation of the nanoscale p-n heterojunctions significantly (by more than four times) enhanced the sensing performance of the fabricated device with a response of 0.62 and 2.6 for pure ZnO and NiO/ZnO heterojunction devices, respectively, which results in excellent LOD of 10 ppb toward ethanol at room temperature [15]. Similar sensing performance was observed for NiO/ZnO nanoscale photodetector upon exposure to UV light, compared to a pure ZnO device [23,35]. 

In addition, the sensor response increases dramatically from 2.6 to 29 by increasing the operating temperature from 30 °C to 150 °C (Figure 14e,f). This excellent sensitivity at the operating temperature of 150 and under light illumination resulted in an exceptional LOD of 2 ppb towards ethanol (Figure 14f), which is the highest sensitivity reported for light-assisted semiconductor-based sensors for ethanol detection [15]. Furthermore, the fabricated ultra-porous device demonstrated an excellent selectivity towards ethanol (15 times higher sensing response) over ethylbenzene (ETBZ), propane (PROP), and acetone (Figure 14g). The negative response toward acetone at room temperature could be attributed to the interaction of the moisture adsorbed on the nanostructured surface with the acetone molecules [15]. However, further investigation is required to provide a better understanding about this negative response from an n-type semiconductor-based sensor (ZnO) toward an oxidizing gas (acetone).

In a similar approach, flame-made Si-doped α-MoO_3_ exhibit excellent sensing properties toward NH_3_ [31]. At an optimum dopant level of 3 wt.% and an operational temperature 400 °C, such sensors showed superior NH_3_ selectivity towards acetone, NO, and CO and accurately detected breath-relevant NH_3_ concentrations down to 400 ppb under 90% RH [31].

## 6. Conclusions

The benefits of non-invasive human breath gas sensors make them an ideal alternative to current standard disease diagnosis methods. These preliminary results are promising and showcase how researchers continually take advantage of low-cost composite nano-dimensional materials for simple human breath gas sensor devices. We hope to see a trend away from studies involving only the use of simulated breath for device validation towards studies primarily validating their sensors via clinical sampling. This is currently a gap in the body of works and presents an opportunity for the next generation of breath sensors to strive towards. Validation by clinical sampling will also result in a greater focus on device integration with researchers needing to meet the expectations of end users.

If current progress precedes the present obstacles preventing human breath gas sensors from entering the commercial multi-billion-dollar biomedical sensor market, such as slow response and high operating temperature, which will be overcome. Therefore, numerous further investigations are required to explore the opportunities available from nano-dimensional materials for human breath gas sensors.
